# Quantification of Pulmonary Fibrosis in a Bleomycin Mouse Model Using Automated Histological Image Analysis

**DOI:** 10.1371/journal.pone.0170561

**Published:** 2017-01-20

**Authors:** Jean-Claude Gilhodes, Yvon Julé, Sebastian Kreuz, Birgit Stierstorfer, Detlef Stiller, Lutz Wollin

**Affiliations:** 1 Biocellvia, Marseille, France; 2 Immunology and Respiratory, Boehringer Ingelheim Pharma GmbH & Co. KG, Biberach, Germany; Helmholtz Zentrum München, GERMANY

## Abstract

Current literature on pulmonary fibrosis induced in animal models highlights the need of an accurate, reliable and reproducible histological quantitative analysis. One of the major limits of histological scoring concerns the fact that it is observer-dependent and consequently subject to variability, which may preclude comparative studies between different laboratories. To achieve a reliable and observer-independent quantification of lung fibrosis we developed an automated software histological image analysis performed from digital image of entire lung sections. This automated analysis was compared to standard evaluation methods with regard to its validation as an end-point measure of fibrosis. Lung fibrosis was induced in mice by intratracheal administration of bleomycin (BLM) at 0.25, 0.5, 0.75 and 1 mg/kg. A detailed characterization of BLM-induced fibrosis was performed 14 days after BLM administration using lung function testing, micro-computed tomography and Ashcroft scoring analysis. Quantification of fibrosis by automated analysis was assessed based on pulmonary tissue density measured from thousands of micro-tiles processed from digital images of entire lung sections. Prior to analysis, large bronchi and vessels were manually excluded from the original images. Measurement of fibrosis has been expressed by two indexes: the mean pulmonary tissue density and the high pulmonary tissue density frequency. We showed that tissue density indexes gave access to a very accurate and reliable quantification of morphological changes induced by BLM even for the lowest concentration used (0.25 mg/kg). A reconstructed 2D-image of the entire lung section at high resolution (3.6 μm/pixel) has been performed from tissue density values allowing the visualization of their distribution throughout fibrotic and non-fibrotic regions. A significant correlation (p<0.0001) was found between automated analysis and the above standard evaluation methods. This correlation establishes automated analysis as a novel end-point measure of BLM-induced lung fibrosis in mice, which will be very valuable for future preclinical drug explorations.

## Introduction

Pulmonary fibrosis is a severe, often progressive pathologic condition in many respiratory diseases with idiopathic pulmonary fibrosis (IPF) being the most common with high morbidity and mortality [1]. Only two treatment options (nintedanib and pirfenidone) were recently approved both showing a partial reduction of the disease progression but no cure or even regression of the disease [2,3]. Hence, the preclinical evaluation of novel compounds in animal models of lung fibrosis represents further on a critical step in drug development. The animal model of BLM-induced lung fibrosis is widely used to characterize the potential inhibitory effect of newly developed drugs. Although this model has important limitations [4] and does only resemble aspects of pulmonary fibrosis in humans it helped also to characterize the currently available drugs for IPF [5,6,7]. Such characterization requires accurate quantification of morphological changes occurring in pulmonary tissue to determine the severity of pulmonary fibrosis and the efficacy of preclinical drugs. Several methods are currently used and considered as end-point measures in BLM-induced lung fibrosis in mice as micro-computed tomography (micro-CT), magnetic resonance imaging (MRI), gene expression analysis, biochemical analysis (hydroxyproline), blood biomarkers and lung function measurements [8–18]. Quantification of the severity of fibrosis is also accessed by means of histological methods based either on grading score [10, 19–24] or by digital imaging analysis [10, 12, 14, 25–27]. Histological scoring has been developed by assigning numerical scores in relationship with the amount of fibrosis in histological samples. Despite the fact that histological scoring is extensively used it does not allow, due to the limited grades of the numerical scale, to achieve a full quantitative analysis of fibrosis. It is noteworthy that the evaluation of scores is observer-dependent and consequently may be subjected to intra- and inter-variability [20]. Histological scoring is performed at a high magnification (x10 or x20) on a limited number of fields (10 to 15) which only accounts for a variable part of the total lung section and is therefore sensitive to bias considering the heterogeneous distribution of fibrosis. Digital imaging analysis is also used to quantify pulmonary morphological changes elicited by BLM-induced fibrosis. Most of the established digital imaging analyses evaluate the severity of BLM-induced fibrosis by quantifying collagen content stained by picrosirius red or to a lesser extent Masson trichrome or even more selectively by immunostaining [12, 14, 25–31]. The expression of collagen is determined from the ratio of the stained area versus the total area of the lung section. The main limitation of histological scoring and digital image analyses is related to the fact that quantitative data refer to an average value of morphological changes of collagen content. Due to the high heterogeneity of pulmonary fibrosis this may result in an underestimation of the severity of fibrosis particularly if analyzing mild pathologies. This may hamper an accurate and reliable evaluation of the efficacy of pharmacologically active compounds.

To overcome this main limitation of grading scores and digital imaging methods we developed a suitable automated software image analysis (automated analysis) based on a new assessment of the pulmonary tissue density from entire histological lung sections. Such a new digital imaging method allowed us to determine the distribution of density values in affected and non-affected alveolar parenchyma regions and then to quantify directly and objectively for the first time the BLM-induced fibrotic alterations. With the aim to establish the automated analysis as a quantitative end-point measure of pulmonary fibrosis in the BLM mouse model, the automated analysis has been compared and correlated to Ashcroft scoring [19], *in vivo* micro-CT analysis and lung function measurements on the same animals. We have provided evidence, from a dose-response study with increasing doses of BLM, that automated analysis allows a robust evaluation of the severity of pulmonary fibrosis. It should be highlighted that automated analysis could be carried out using a single dose administration of BLM, at a lower concentration than those usually used in most bleomycin studies. The lower dose of BLM reduces the stress for the animals used and increases the chance for successful and reliable drug testing.

## Materials and Methods

### Animals

Eight to 12 week-old C57/BL6 male mice (n = 54) (Charles River, Sulzfeld, Germany), 22 to 30 g, were used throughout the study. Animal husbandry and experimentation was conducted in accordance with German national guidelines and legal regulations and the guidelines of the Association for Accreditation of Laboratory Animal Care. The permission for the animal experiments was issued by the Regierungspräsidium Tübingen, Germany.

### BLM-induced pulmonary fibrosis

To facilitate BLM administration animals were anaesthetized for a short period of time using inhaled isoflurane (3–5%). A single dose of BLM (Calbiochem, Darmstadt, Germany) 0.25, 0.5, 0.75 and 1 mg/kg (1–4 U/mg) (n = 12 mice per group) in sterile isotonic saline (50 μL per animal) was intratracheally instilled by means of a 22 gauge plastic cannula (Vasofix, 0.5x25 mm, B. Braun Melsungen, Germany) coupled to a 1 mL syringe to each animal at the start of the study (day 0). The same volume of sterile saline was administered to control mice (n = 6 mice). Body weights were monitored throughout the whole experiment. Non of the animals dropped out of the study due to weight loss till the end of the study at day 14.

### Lung function measurement

On day 14 mice were anesthetized with pentobarbital 48 mg/kg combined with xylazine 2.32 mg/kg injected i.p.. After tracheotomy and intubation, a tracheal cannula was connected to a FlexiVent system (SCIREQ, Montreal, PQ, Canada) for pulmonary function testing. To prevent spontaneous breathing the animals received pancuronium bromide 0.64 mg/kg i.v. (Inresa Arzneimittel GmbH, Freiburg, Germany). Ventilation was conducted with 150 breath/min, a tidal volume of 10 mL/kg and an end-expiratory pressure of 3 cm H_2_O. After recruiting total lung capacity (30 cm H_2_O) dynamic lung compliance (Cdyn) and forced vital capacity (FVC) was measured.

### In vivo micro-CT analysis

Fourteen days after BLM administration *in vivo* micro-CT analysis of the whole lung was performed by means of a volumetric assessment of BLM-induced changes in x-ray absorption. Animals were anesthetized with isoflurane 1.5% and fixed in prone position. Micro-CT images were acquired on a Quantum FX micro-CT system (Perkin Elmer, Waltham, MA) with cardiac gating (without respiratory gating), using the following parameters: 90 kV; 160 μA; FOV, 60 x 60 x 60 mm; spatial resolution, 0.11 mm, resulting in a total acquisition time of 4–5 minutes. Resulting images were analyzed with MicroView 2.0 software (GE Healthcare, Amersham, UK), which allows a semi-automatic segmentation of left and right lungs. Hounsfield unit (HU) histograms were obtained for left and right lungs using bins of 10-HU width. In the absence of a well-established gold standard to quantify fibrotic changes in rodents with micro-CT and to avoid relying on an arbitrary threshold to identify fibrotic regions [14,15] the HU corresponding to the peak of the HU-histogram for the segmented pixels were used as a measure of the progression of fibrosis. Total lung volume was also computed.

### Histopathology score

Following micro-CT analysis and lung function measurements the animals were euthanized at day 14 by an overdose of pentobarbital i.p., lungs were excised and lung wet weight was determined. After cannulation of the trachea the lungs were inflated with paraformaldehyde 4% (PFA) for 20 min at a pressure of 20 cm H_2_O. The filled lung was then sealed by a ligature and immersed in PFA for at least 24 h. Subsequently PFA fixed lungs were embedded in paraffin following standard procedures. Three serial slices of 3 μm thickness each 50 μm apart of both lung lobes were stained with Masson Trichrome. Fibrotic scoring was performed by a blinded pathologist according to the scale defined by Ashcroft [19]. The slices were analyzed with a microscope using an x10 objective.

### Automated histological image analysis (automated analysis)

**Fig 1:**
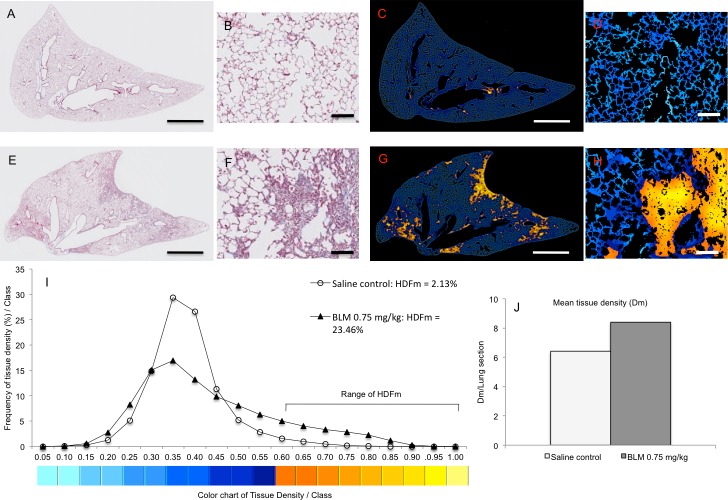
Representative images of original and 2D-reconstructed lung sections for the quantification and the mapping of pulmonary tissue density by means of digital automatic analysis. Masson trichrome-stained images (A, E) and their respective 2D-reconstructed images (C, G) correspond to a lung section from saline control (A, C) and BLM-treated (0.75 mg/kg) lungs (E, G). Panels B, D, F, H show details from their original stained (B, F) and 2D-reconstructed (D, H) images at higher magnification. Lung tissue density was determined from thousands of micro-tiles crisscrossing entire lung sections. For mapping thousands tissue density values throughout lung section density values were graded in 20 classes of increasing values (I) and pseudocolours were assigned from blue (low density values) to yellow (high density values) according to their classification. Note that high density values (yellow) were restricted in alveolar parenchyma of BLM-treated lung (G, H) and located in fibrotic lesions evidenced in the respective original stained image (E, F). The frequency of tissue density (I) was determined from the classification of the whole unitary density values obtained in each lung section (A, E). HDFm index corresponds to the sum of the frequencies of the highest tissue density (classes 12 to 20) expressed in fibrotic foci. The mean tissue density (Dm) (J) was evaluated for each lung section from thousands of micro-tiles. Scale bars: 1 mm (A, C, E, G), 100 μm (B, D, F, H).

The total sets of lung slices used for the above standard fibrosis evaluation were scanned at x20 magnification using a NanoZoomer-SQ and digital images of entire lung sections were captured using the NDP.view 2 software (both from Hamamatsu Corporation, Hamamatsu, Japan). To focus the analysis on alveolar parenchyma the walls of large bronchi located in the vicinity of the lung lobes, small bronchi and vessels (diameter >200 μm) associated with alveolar parenchyma as well as their surrounding collagen fibers were manually deleted using Gimp 2.8 software (Free Software Foundation Inc.). Digital images were then reduced from x20 to x2.5 magnification with a pixel size of 3.632 μm allowing both a high-resolution visualization of morphological structure of pulmonary tissue and a very short processing time for the software analysis (<1 sec per entire lung section). The quantification of the BLM-induced fibrotic alterations has been assessed on the basis of pulmonary tissue density. For this purpose Biocellvia has developed a proprietary software program allowing the determination of pulmonary tissue density from thousands of micro-tiles (30–56 μm^2^) crisscrossing the selected pulmonary tissue of entire lung sections. The density of the pulmonary tissue was evaluated for all individual micro-tiles corresponding to the ratio of the area of the lung tissue inside the micro-tile and the total area of the micro-tile. To quantify the distribution of pulmonary tissue densities they were graded in 20 classes of increasing values in increments of 0.05 (Fig 1). The frequency of tissue density was calculated dividing the number of tissue density values in a given class by the total number of density values in all 20 classes. A distribution of the frequency of tissue density according to their classification was then determined for a comparison between saline-treated control lungs and BLM-treated lungs. To visualize the distribution of density values 2D-reconstructed images of lung sections were composed by assigning pseudocolors to tissue density values according to their classification (Fig 1). These 2D-reconstructions were compared with the Masson trichrome-stained slices.

Two pulmonary tissue density indexes were defined with the aim to quantify and compare pulmonary fibrotic alterations in saline-treated control lungs and BLM-treated groups: (i) the mean tissue density (Dm) and (ii) the high tissue density frequency (HDFm). Dm corresponds to the mean density value of alveolar parenchyma determined from individual density values of the micro-tiles. Dm can be expressed per lung section, per animal or per group. HDFm was established to specifically quantify fibrotic alterations in alveolar parenchyma. HDFm corresponds to the sum of frequencies of high tissue densities limited to fibrotic alterations. The range of classes allocated to HDFm was established from the saline-treated control group wherein fibrotic alterations were missing and for which high tissue densities frequency per class were less than 1%. In the present study HDFm corresponded to the sum of frequencies ranging from class 12 to 20 (Fig 1).

### Statistical analysis

Data are presented as mean ± standard error of mean. Statistical differences between groups were analyzed by one-way analysis of variance (ANOVA) with subsequent Dunnett’s multiple comparison test for all parametric data and Kruskal-Wallis test followed by Dunn’s multiple comparison test for nonparametric data (GraphPad Prism 6.0; GraphPad Software, Inc. La Jolla, CA), Spearman correlation coefficient has been calculated to determine the statistical significance of correlated data. A p-value <0.05 was considered statistically significant.

## Results

### Quantification of lung fibrosis by automatic histological analysis

Histological lung sections stained with Masson trichrome were scanned and high resolution digital images (3.632 μm/pixel) of entire lung sections were obtained. Following a manual selection of alveolar parenchyma automatic histological analysis was applied to assess the mean pulmonary tissue density (Dm) in the saline-treated control lungs and BLM-treated lungs. Dm was evaluated from thousands of micro-tiles (30–56 μm^2^) crisscrossing the entire pulmonary tissue of digital images of each lung section.

**Fig 2:**
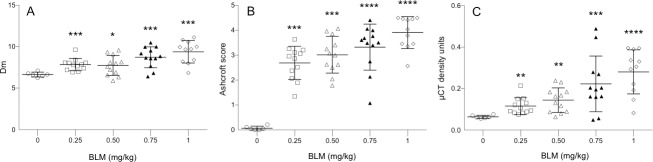
Bleomycin (BLM) administration resulted in elevated lung tissue density and fibrotic Ashcroft scoring. 14 days after intratracheal BLM administration at doses of 0.25, 0.5, 0.75 and 1.0 mg/kg lung tissue alterations was assessed on the same whole lung slices stained with Masson trichrome by automatic histological analysis (A) and Ashcroft scoring (B) and in vivo in the same animals by means of micro-CT analysis (C). BLM-treated mice (n = 12/dosing group) were compared to saline control mice (n = 6). Data are shown as mean ± s.e.m. The mean tissue density (Dm) determined by automatic histological analysis (A) was assessed from thousands of micro-tiles covering the whole lung sections of each group of mice. *p<0.05, **p<0.01, ***p<0.001, ****p<0.0001 was considered statistically significant in comparison to the non BLM-treated control group. ns: non-significant.

**Fig 3:**
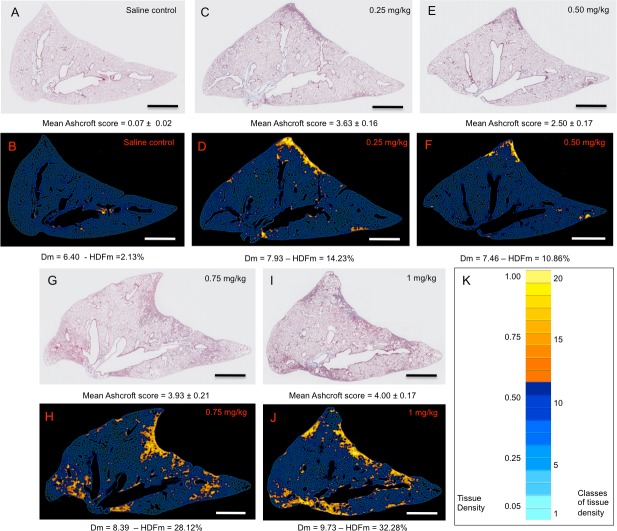
Representative original images of Masson trichrome stained lung sections and their corresponding automated 2D-reconstructed images according to their respective lung tissue density values. Original images (A, C, E, G, I) correspond to representative Masson trichrome stained lung sections from saline control (A) and BLM-treated lungs at 0.25 mg/kg (C), 0.50 mg/kg (E), 0.75 mg/kg (G) and 1 mg/kg (I). Reconstructed-2D lung sections of their respective original images were obtained by grading their tissue density values, obtained from thousands micro-tiles, in 20 classes of increasing values. Pseudocolours were then assigned to tissue densities according to their class from light blue (low density values) to yellow (high density values) (K). For each original image of saline control and BLM-treated lungs the value of their Ascroft score (mean ± s.e.m.) was indicated. Regarding their corresponding 2D-reconstructed images the value of their Dm (mean ± s.e.m.) and HDFm was indicated, respectively. Note that high density values visualized in 2D-reconstructed images were focalized in fibrotic regions evidenced in their corresponding Masson trichrome stained lung sections. Scale bars: 1 mm.

At day 14, BLM induced a dose-dependent statistical significant increase of Dm in all BLM-treated lungs compared to control lungs (Fig 2A). The observed increase of Dm were in agreement with pathological alterations of lung structures 14 days after administration of BLM in particular thickening of alveolar walls, formation of fibrous bands and fibrous foci (Figs 1 and 3). Such pathological alterations were extensively described in various mouse models of BLM-induced lung fibrosis [4, 8, 19, 20, 22]. It is noteworthy that no statistically significant differences in Dm were found between the BLM-treated lungs of the 0.25 mg/kg and 0.5 mg/kg group and between the 0.75 mg/kg and 1.0 mg/kg group.

With the aim to map the topographical distribution of fibrosis, 2D-reconstructed images of entire lung sections were composed on the basis of their tissue density values determined from thousands individual micro-tiles. For this purpose pulmonary tissue densities were graded in 20 classes of increasing values in increments of 0.05 and pseudocolours were assigned from blue (lower density values) to yellow (higher density values) according to their classification (Figs 1 and 3). The mapping of pulmonary tissue density in saline-control lung sections was characterized essentially by low tissue density values (Figs 1 and 3). In lung sections of BLM-treated groups in addition to low density values we observed the presence of high tissue density values which were specifically located in fibrotic regions as evidenced by comparing Masson trichrome images with their respective 2D-reconstructed images (Figs 1 and 3). These fibrotic regions were characterized by small to large fibrotic masses with progressive obliteration of alveolar septa. According to the scale defined by Ascroft et al [19] and Hübner et al [20] these fibrotic regions belonged to elevated scores ranging from 5 to 8.

**Fig 4:**
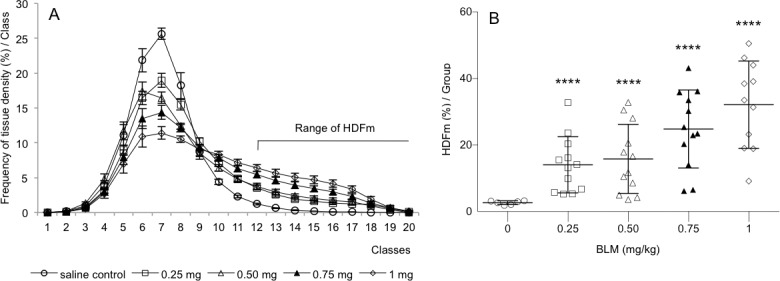
Distribution of tissue density frequency and determination of high tissue density frequency (HDFm) in saline control and bleomycin (BLM)-treated lungs. Lung tissue density was determined from several thousands of micro-tiles covering the whole lung sections of saline control (n = 6) and BLM-treated (n = 12/dosing group) mice. A: Tissue density values were graded in 20 classes of increasing values (mean ± s.e.m) and their frequency per class was expressed in percent (compared to the total number of density values). B: BLM administration induced a dose-dependent increase of HDFm which corresponded to the sum of the percentage of tissue densities from class 12 to 20. ****p<0.0001 was considered statistically significant in comparison to the non BLM-treated control group.

With the aim to quantify specifically fibrotic lesions in alveolar parenchyma HDFm index was assessed in saline-treated control and BLM-treated lungs. HDFm corresponds to the sum of frequencies of high tissue densities expressed specifically in BLM-treated lungs (Figs 1 and 3). Under these conditions the range of HDFm classes was established from the distribution of the frequency of tissue density in the saline-treated control group. In the present study the threshold of the range was set up at class 12 for which high density frequency value was below 1% (Figs 1, 3 and 4). HDFm value corresponded to the sum of the frequencies of high density values ranged from class 12 to 20. The 2D-reconstructed images of saline control lungs revealed that density values ranged from class 12 to 20 were not related to fibrotic alterations but to a noise background formed by some remaining collagen fibers and/or erythrocytes which were not completely deleted from the manually selection of the alveolar parenchyma (Figs 1 and 3). At day 14, BLM induced a dose-dependent statistical significant increase (p<0.0001) of HDFm in all BLM-treated lungs compared to control lungs (Fig 4B). These quantitative data were in line with those assessed by the Dm index. HDFm turned out to be a greater discriminator of pulmonary fibrosis than Dm. This notion is supported if the percentage of the increase of Dm and HDFm between saline control and BLM-treated lungs is compared. For Dm the BLM-induced increase ranged between 18% to 41% (Fig 2A) but for HDFm between 425% and 1100% (Fig 4B). As expected BLM administration induced fibrotic changes in the alveolar parenchyma resulting in a marked decrease of the percentages of lower frequencies of tissue density and in parallel a marked increase of the higher frequencies. Such changes in the distribution of tissue density elicited by fibrotic alteration of the alveolar parenchyma were accurately quantified by Dm and HDFm indexes independent of the BLM concentration used (Fig 4).

### Validation of automatic histological analysis in mice lung fibrosis

In order to define the potential value of Dm and HDFm indexes as outcome measures in preclinical studies of lung fibrosis we have explored their correlation with established standards namely Ashcroft scoring, micro-CT imaging and lung function measurements.

**Fig 5:**
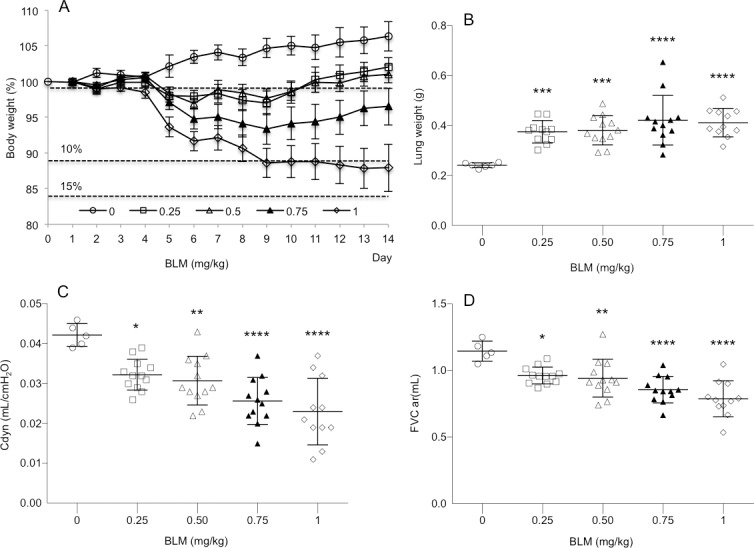
Bleomycin (BLM) administration resulted in a reduced body weight gain and increased lung wet weight and in parallel in impaired lung functions. 14 days after intratracheal BLM administration at doses of 0.25, 0.5, 0.75 and 1.0 mg/kg the mean ± s.e.m. body weight (A), lung wet weight (B), dynamic lung compliance (Cdyn) (C) and force vital capacity (FVC) (D) were determined in saline control (n = 6) and BLM-treated (n = 12/dosing group) mice. *p<0.05, **p<0.01, ***p<0.001, ****p<0.0001 was considered statistically significant in comparison to the non bleomycin-treated control group.

The severity of the BLM-induced alterations was examined by comparing the body weight and the lung wet weight of saline-treated control mice with BLM-treated (Fig 5A and 5B). At day 14, BLM at the concentration of 0.75 and 1 mg/kg caused a statistical significant reduction of the body weight gain during the 14 day experimentation period by 6 and 12%, respectively. No significant body weight reduction was observed at the lower BLM concentrations 0.25 and 0.50 mg/kg. Already the lowest dose of 0.25 mg/kg BLM induced a statistical significant increase (p<0.001) of the lung wet weight by 56% (Fig 5B). A dose-dependent increase of lung wet weight was observed for higher BLM concentrations 0.5, 0.75 and 1.0 mg/kg corresponding to 58, 75 and 71% increase, respectively. Such increase of lung wet weight in BLM-treated mice was in agreement with histopathological observations and Ashcroft scoring analysis we performed in Masson trichrome stained lung sections obtained from the same mice. Independent from the BLM dose used all lungs from BLM-treated animals showed peribronchial and subpleural fibrosis. BLM administration induced a dose-dependent statistically significant increase in the Ashcroft scores (Fig 2B).

**Fig 6:**
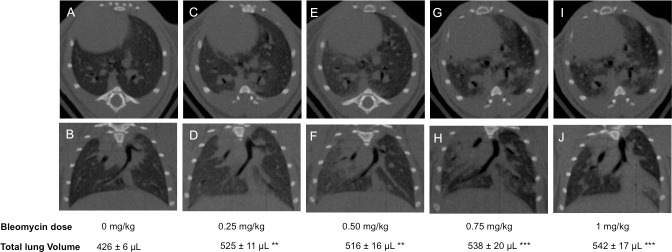
Representative micro-CT images of lung sections from saline control and bleomycin-treated mice. Transverse (top row) and corresponding coronal (bottom row) micro-CT images acquired at 14 days after saline (control) (A, B) and bleomycin administration at the concentration of 0.25 mg/kg (C, D), 0.50 mg/kg (E, F), 0.75 mg/kg (G, H) and 1mg/kg (I, J). Total lung volume is given as mean ± s.e.m. (n = 12/dosing group). **p<0.01, ***p<0.001 was considered statistically significant in comparison to the non bleomycin-treated control group.

BLM administration induced a dose-dependent increase in lung tissue density assessed by micro-CT analysis at day 14 (Figs 2C and 6). At a dose of 0.25, 0.5, 0.75 and 1.0 mg/kg BLM-induced a statistically significant increase of lung tissue density by 18.3% (p<0.05), 133% (p<0.05), 267% (p<0.01) and 366% (p<0.0001), respectively.

At day 14, BLM administration induced a dose-dependent statistically significant decrease of dynamic lung compliance (Fig 5C) and forced vital capacity (Fig 5D). Even at the lowest BLM dose of 0.25 mg/kg significant differences were detected compared to the control group.

**Fig 7:**
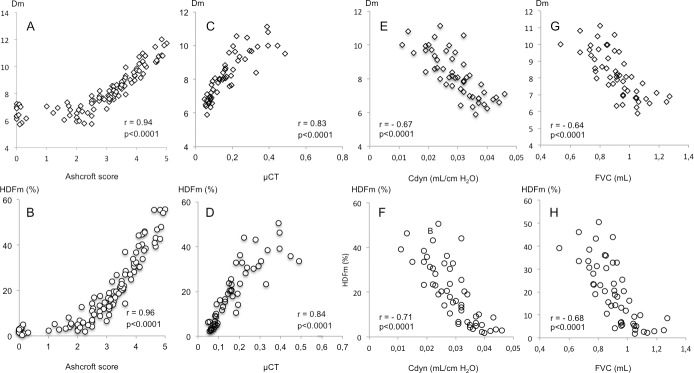
Correlation of tissue density (Dm) and high frequency density (HDFm) indexes with Ashcroft score, micro-CT and lung function measurements in the bleomycin mice model. The agreement of the Dm and HDFm measurements with those obtained with Ashcroft scoring (A, B), micro-CT analysis (C, D), dynamic lung compliance (Cdyn) (E, F) and force vital capacity (FVC) (G, H) was evaluated using linear regression analysis. Data correspond to mean values per animal of saline control (n = 6) and BLM-treated (0.25, 0.50, 0.75, 1 mg/kg) (n = 12/dosing group) mice. r = Spearman correlation coefficient.

The correlation between the Dm and HDFm indexes with Ashcroft scoring, micro-CT analysis and lung function was evaluated using linear regression analysis (Fig 7). Dm and HDFm mean values demonstrated good correlation with Ascroft score mean values (Dm: r = 0.94, p<0.0001, HDFm: r = 0.96, p<0.0001), mean tissue density values assessed by micro-CT (Dm: r = 0.83, p<0.0001, HDFm: r = 0.84, p<0.0001), dynamic lung compliance (Dm: r = - 0.67, p<0.0001, HDFm: r = - 0.71, p<0.0001) and force vital capacity (Dm: r = - 0.64, p<0.0001, HDFm: r = - 0.68, p<0.0001).

## Discussion

The present study has shown that the automated histological image analysis (automated analysis) allowed a robust histological evaluation of pulmonary fibrosis in the BLM mouse model. Automated analysis was performed by assessing lung tissue density determined from thousands of microtiles crisscrossing sections from the entire lung. With the aim to quantify BLM-induced lung fibrosis two tissue density indexes have been established from individual tissue density values: the mean tissue density (Dm) and the high tissue density frequency (HDFm). In addition, pseudocoloured 2D-reconstructed images of lung slices were composed reflecting the tissue density values. These 2D-reconstructed images enabled visualization and mapping of the distribution of tissue density values in fibrotic and non-fibrotic regions of the lung slices and allowed to select target regions (e.g. fibrotic foci). The potential value of both density indexes as an outcome measure of the severity of lung fibrotic alterations has been validated by their correlation with current standard histological scoring (Ashcroft scores), *in vivo* imaging technique (micro-CT) and lung function testing.

Because the main bronchi, vascular walls and their surrounding collagen fibers were removed before the images were analyzed the morphological changes assessed by Dm and HDFm indexes were related to fibrotic alterations restricted to the alveolar parenchyma. The Dm index provided access to the mean value of morphological changes occurring in the entire lung sections whereas HDFm index to the mean value of fibrotic alterations. Contrary to the HDFm the Dm index reflects the distribution of fibrotic and non-fibrotic regions in lung sections and may underestimate the percentage of small fibrotic alterations induced either by low BLM concentrations or pharmacological interventions. Although Dm index was less discriminating that HDFm index it allowed to quantify accurately small morphological changes induced by a single dose of BLM (0.25 mg/kg) a concentration lower than the usual dose in rodents [8].

In the present study we sought to validate Dm and HDFm indexes as a direct read out of morphological changes induced in the BLM mouse model comparable to that currently performed by means of established histological scoring methods [19, 20]. The automated analysis and Ashcroft scoring methods were carried out on the same histological sections stained with Masson trichrome to include the changes in collagen in the overall quantification of the morphological changes in the lung. Collagen plays an important role in the fibrotic remodeling of the alveolar parenchyma and accordingly its quantification is frequently assessed by hydroxyproline assays or quantification using picrosirius red or Masson trichrome staining. However, in the present study hydroxyproline method would not allow to pick up subtle fibrotic alterations induced in the alveolar parenchyma [25]. Considering the available histological digital imaging methods they allow an overall quantification of collagen content in the alveolar parenchyma, bronchi and vessels [12, 14] but would not allow to quantify the distribution of collagen content restricted to fibrous and non-fibrous regions of the alveolar parenchyma in view of its correlation with density indexes.

A substantial portion of the quantitative histological analyses of fibrotic lung alterations is still studied by means of scoring methods [19, 20, 22, 32]. The comparison between the automated analysis and the Ashcroft scoring on the same lung sections has led to a highly statistical significant correlation which contributes to validate Dm and HDFm indexes as a direct read out of morphological changes in lung tissue. However, Ashcroft scoring, as well as histological scoring, has major limitations that were not exhibited by automated analysis. Histological scoring is not observer-independent and then subject to intra- and inter-variability [20]. Histological scoring is in general not performed on the entire lung section but on a limited number of microscopy fields, which did not account for the total lung section area and therefore is sensitive to variability considering the heterogeneous distribution of fibrosis. These limitations may also restrict comparative studies between different laboratories.

A highly significant correlation has been established between the automated analysis and the *in vivo* micro-CT analysis which, in addition with Ashcroft scoring, contributes to validate automated analysis as an end-point measure for quantifying the severity of BLM-induced pulmonary fibrotic alterations in mice. It is noteworthy that i*n vivo* micro-CT analysis is currently one of the key investigative methods for studies exploring pulmonary morphological changes longitudinally [4,8,20,32,33]. Magnetic resonance imaging (MRI) is also a valuable technique in the longitudinal follow-up of fibrosis progression in BLM mouse models [10,12,14–16] which might be used in future studies as a complementary approach to the present *ex vivo* automated analysis. In a recent study it was demonstrated that data obtained with micro-CT analysis are in agreement with those obtained by MRI analysis [16]. Assessment of the pulmonary tissue density by automated analysis and micro-CT requires very different software programs and imaging procedures to achieve the objectives. Accordingly, marked differences have been observed notably for resolution and image acquisition time. *In vivo* micro-CT resolutions are usually in the range of 25–90 μm/pixel considering multiple constrains due to respiratory and cardiac motion and scanning/reconstruction time [11,34–37]. Hence, *in vivo* micro-CT resolutions are much lower compared to the high resolution (3.5 μm/pixel) used in the automated histological analysis. An improved micro-CT analysis using higher sensitivity involving high resolution scanning [13,34,38,39] might also be used to enhance accurate quantification of low level of fibrotic alterations. High resolutions (1–2 μm/pixel) have also been achieved with micro-CT analysis on *ex vivo* fixed lungs [34]. However, such resolutions can only be applied on restricted 3D fields of some millimeters. In contrast, the automated analysis is applied to the entire lung section. It should be noted that micro-CT high resolutions are associated with sometimes prohibitively long scan/reconstruction times of several hours per sample precluding high throughput [13,34]. In contrast the automated analysis acquisition/analysis time is much shorter in the range of 1 hour for 100 entire lung sections. *Ex vivo* micro-CT may also be used for a 3D evaluation of severity of lung fibrosis providing a more sensitive and quantitative measure as well as a marked reduction of scanning time [13].

A potential limitation of the automated analysis approach is related to the exclusion of the large bronchi and vessels manually before starting the automated image processing. Such preparation is simple and requires less than 3 hours for 100 lung sections. However, the manual exclusion of large bronchi and vessels might be subject to bias in their delimitation that might impact on the assessment of Dm index. Nevertheless, considering the elevated surface of lung tissue analyzed per group such impact can be considered insignificant. We also underlined that manual exclusion did not interfere on the evaluation of HDFm index since it was determined exclusively from fibrotic alterations located in the alveolar parenchyma. The manual exclusion of large bronchi and vessels from the automated analysis limits the use of the automated analysis to pharmacological principles not affecting bronchi and large vessels.

In conclusion, the presented automated analysis of images of lung sections from mice treated with bleomycin represents a fully accurate and reliable quantification of bleomycin-induced fibrotic lung alterations. The sensitivity to quantify precisely the fibrotic lung alterations at low bleomycin concentrations is a major improvement. It should be highlighted that using low BLM concentration may be very attractive for future studies since it allows to test the efficacy of compounds in animals in much better shape, eliminating the morbidity, and contributing therefore to a higher reliability of the preclinical drug evaluation. The high correlation between the newly developed automated analysis and standard evaluation methods like Ashcroft scoring, micro-CT and lung function testing establishes it as a new, reliable, accurate and fast alternative to determine fibrotic lung alterations. In line with micro-CT, automated image analysis may be regarded as a new additional or complementary robust measurement for future preclinical drug testing.
